# A methodological framework for developing bioinformatics databases: a case study on the *Boesenbergia rotunda* genome database

**DOI:** 10.3389/fgene.2026.1901079

**Published:** 2026-09-09

**Authors:** Sarinder Kaur Dhillon, Wai Shi Pang, Chee How Teo, V. R. Naresh Mutha, Jennifer Ann Harikrishna

**Affiliations:** 1 Institute of Biological Sciences, Faculty of Science, University of Malaya, Kuala Lumpur, Malaysia; 2 Centre for Research in Biotechnology for Agriculture, University of Malaya, Kuala Lumpur, Malaysia; 3 Department of Genetics, Cell Biology and Anatomy, University of Nebraska Medical Center, Omaha, NE, United States

**Keywords:** bioinformatics, database, genomics, medicinal plant, plant genomics

## Abstract

Comprehensive, well-structured databases are critical for storing, integrating, and analyzing genomics data. This manuscript presents a hybrid methodology combining the traditional Database Development Life Cycle (DDLC) with agile principles to build a comprehensive genome database for *Boesenbergia rotunda*, known as Fingerroot ginger. *Boesenbergia rotunda* (L.) Mansf., also recognized as Fingerroot ginger or Chinese keys, is a perennial herb from the Zingiberaceae family in the order Zingiberales. Fingerroot ginger is widely used in Asian cuisine, especially the rhizome, and is recognized for its potent bioactive compounds, including panduratin A, 4-hydroxypanduratin, and cardamonin, which are reported to have notable anti-inflammatory, anti-tumor, and antimicrobial, especially antiviral, effects. The Fingerroot Genome Database incorporates genome, transcriptome, coding sequences, and functional annotations in a relational schema of 27 tables. Using iterative refinement during modular development, we integrated tools such as BLAST+ and JBrowse2 to support sequence search and genome visualization. This case study illustrates how a structured DDLC approach can be combined with Agile-inspired refinement to guide the development of a species-specific bioinformatics database for an underexplored medicinal plant.

## Introduction

The proliferation of high-throughput sequencing technologies has led to an unprecedented surge in genomic data, necessitating structured repositories tailored to individual species. Bioinformatics databases provide a centralized platform for storing, retrieving, and analyzing such complex data (Dhillon, 2019). With the current advancement of technologies which includes artificial intelligence, database development has become much more important especially for data curation and analysis. Data that is not stored in digital platform is a hindrance to adoption of new technologies. However, traditional database development models are often too rigid for biological data, which evolves with new discoveries and size. Many bioinformatics databases fail as the development itself did not follow proper standards or database development methods.

This manuscript proposes a hybrid methodology combining traditional plan driven Database Development Life Cycle (DDLC) phases with agile software development to address this gap, using the Fingerroot Genome Database as a case study. We explain the development of a comprehensive genome database combining data integration, functional annotation, and user-accessible interfaces. We also present a use case for the database and embedded tools. The hybrid approach used in this study merges the structured phases of the Database Development Life Cycle (DDLC) with the flexibility of Agile development methodology. The DDLC offers a systematic framework that ensures rigorous data management, integrity, and scalability, which is essential for bioinformatics applications.

## Database development process

Databases form the foundation of modern information systems, supporting data storage, retrieval, and management across industries. The design and development of databases require systematic approaches to guarantee data integrity, efficiency, and adaptability to evolving requirements. Database development methodologies provide the necessary frameworks to guide this process, ensuring that stakeholders’ needs are addressed while balancing constraints such as cost, time, and system performance. The System Development Life Cycle (SDLC) is a widely adopted structured methodology that typically consists of several phases, including planning, analysis, design, implementation, testing, deployment, and maintenance. These phases can be adapted to database development through requirements analysis, conceptual design, logical design, physical design, implementation, testing, deployment, and maintenance or evolution. The SDLC is often integrated with other methodologies to provide a comprehensive framework for database development.

### Traditional methodologies (plan-driven)

#### Waterfall methodology

The waterfall model represents a linear, sequential approach to database development. The stages which include requirements gathering, design, implementation, testing, deployment, and maintenance are executed in order, with little scope for iteration. This methodology is suitable for projects with stable requirements as it is well structured with clear documentation. However due to this, it can be inflexible, costly to adapt to changes and not suitable for dynamic environments.

#### Spiral methodology

The spiral model integrates iterative development with risk management. Development proceeds in cycles (or “spirals”), each consisting of planning, risk assessment, engineering, and evaluation. It is effective for large and high-risk projects; emphasizes risk reduction but it can be resource-intensive and requires advanced project management.

### Iterative and user-centered methodologies

#### Prototyping methodology

Prototyping involves creating a preliminary working model of the database system, which is refined based on user feedback. It encourages stakeholder involvement and useful when requirements are unclear but may result in incomplete documentation and prone to scope creep.

#### Rapid application development (RAD)

RAD emphasizes quick development through iterative prototyping, user participation, and the use of computer-aided software engineering (CASE) tools. It accelerates delivery, fosters continuous feedback but may compromise scalability and long-term performance.

### Contemporary methodologies

#### Agile methodology

Agile methodologies adopt an incremental and iterative approach, dividing development into short sprints. The database evolves alongside application components, with regular stakeholder feedback. This methodology fosters high adaptability, rapid delivery, continuous collaboration but requires intensive stakeholder engagement; may reduce emphasis on documentation.

#### Top-down and bottom-up approaches

Top-Down approach begins with a conceptual design (e.g., entity-relationship diagrams) before moving to logical and physical models whereas bottom up starts with individual data attributes, gradually building entities and relationships. Hybrid approaches are often used in practice to combine the strengths of both.

### Existing related databases and tools

There are abundant databases for plant genomes, Table 1 provides some selected examples that are related to our work.

**TABLE 1:** Comparison of related databases and tools.

Database	Focus species/Scope	Main strengths	Unique tools & features	References
CuGenDBv2(Cucurbit Genomics Database)	Cucurbits (*Cucumis*, *Citrullus*, *Lagenaria*, etc.)	Hosts 34 reference genomes from 27 species; strong comparative genomics focus	Expression atlas, genetic variation data, synteny viewers, breeding-oriented tools	Yu et al. (2023)
Sol Genomics Network (SGN)	Solanaceae (*Solanum lycopersicum*, pepper, potato, etc.)	Latest tomato genome (SL4.0), breeder tools, rich phenotypic data	SolBrowse genome viewer, comparative map viewer, pathway databases, breeder-focused germplasm tools	Fernandez-Pozo N et al. (2015)
Banana Genome Hub	*Musa* spp. (banana and plantain)	Pangenome and multi-omics integration	Synteny visualization, variant catalogs, pangenome graph explorer, differential expression analysis	Droc et al. (2013)
RAP-DB (Rice Annotation Project Database)	*Oryza sativa* (japonica cv. Nipponbare)	High-quality, manually curated gene models	Functional annotation, integration with Gramene and rice research networks	Kawahara, Y. et al. (2026)
Phytozome Next (DOE JGI)	Broad plant coverage (model and crop species across many families)	Comparative genomics across diverse lineages; phylogenetic-based gene family building	PhytoMine query system, JGI Plant Gene Atlas (expression), ortholog/paralog tracing, lineage-specific gene evolution	Goodstein, D. M. et al. (2012)
Fingerroot Genome Database	*Boesenbergia rotunda* (L.) Mansf	Encompasses a comprehensive repository of genetic information, featuring a total of 10,627 assembled genome contig sequences and 95,847 assembled transcriptome sequences for *B. rotunda*. Each genome contig is uniquely labelled with a contig ID, while each transcriptome sequence is assigned a distinct transcript ID. It also houses 65,535 sequences annotated as tandem repeats, along with Transposons and Transposable Elements (TEs) identified using Repeat Masker and Protein. Database provides information on the 73,102 CDSs, along with its corresponding translated protein sequence Repeat Mask. Annotated information on non-coding RNA is also provided, including details on 213 microRNA (miRNA), 3173 transfer RNA (tRNA), 486 ribosomal RNA (rRNA), and 2136 small nuclear RNA (snRNA) genes	BLAST+ and JBrowse2	This study

## Materials and methods

In this section the key Database Development Life Cycle methods are explained. Initially a feasibility study was conducted. This phase involved assessing project scope, available data resources, technological requirements, and consultation with domain experts to ensure alignment with bioinformatics goals. Functional and non-functional requirements were gathered, including support for genome and transcriptome data integration, multi-format annotation, search capability, and web-based tools like BLAST and genome browsersSubsequently, in the design phase, an Entity Relationhsip (ER) diagram was constructed to define the relational structure of the database. Schema normalization ensured minimal redundancy and optimized performance. The schema was divided into modules: genome, transcriptome, CDS, annotations, and BLAST mappings. In the implementation stage, MySQL was used for backend construction. Raw data from 27 CSV files were imported, indexed, and cross-linked. The LAMP stack supported web deployment. Interfaces were developed iteratively in sprints using HTML, CSS, and JavaScript (available at https://doi.org/10.6084/m9.figshare.31957764). Each module underwent unit testing to verify data accuracy (e.g., mapping gene names to annotations). Integration testing ensured consistency between genome-transcriptome mappings and annotation layers. Finally, the database was deployed on a Linux server on the cloud environment with secured access. Tools such as SequenceServer and JBrowse2 were installed and configured for performance. Versioning and backup mechanisms were included for reliability. Agile methodology principles enhanced the development through incremental sprints with well-defined goals (e.g., integrating protein annotations or adding visualization tools), continuous feedback cycles with project stakeholders and domain researchers, adaptive planning to incorporate new datasets or feature requests without disrupting ongoing development and emphasis on deliverable functionality and usability over rigid planning.

The following sections contains detailed methodology on the development of the *Boesenbergia rotunda* Genome Database.

## Implementation timeline and iterative refinement

The development of the Fingerroot Genome Database was conducted as a case-study implementation using a hybrid Database Development Life Cycle (DDLC) and Agile-inspired approach. The DDLC phases provided the overall structure for feasibility assessment, requirements analysis, schema design, implementation, testing, deployment, and maintenance, while Agile principles were applied during implementation through iterative refinement of database modules and user-facing functions.

The project began with an initial feasibility and requirements analysis phase, during which the available genome, transcriptome, coding sequence, annotation, and gene-mapping datasets were reviewed. This was followed by conceptual and logical database design, including the construction of the entity relationship model and the organization of data into relational tables. 1mplementation was then divided into functional modules, including genome data import, transcriptome data import, CDS integration, annotation tables, gene name mapping, query interface development, BLAST integration, and genome browser integration.

Several requirements evolved during development. For example, the initial focus was on storing genome and transcriptome records with their annotations. However, feedback from domain researchers indicated that users would need to search the database using biologically meaningful terms such as gene names, rather than only internal sequence identifiers. As a result, gene name mapping using UniProt-derived accession information was added as an additional requirement. Similarly, the need to support exploratory sequence comparison led to the integration of BLAST through SequenceServer. The requirement for visual inspection of genomic regions and annotation tracks later motivated the integration of JBrowse2. These changes were incorporated without restructuring the whole database because the relational schema and modular implementation allowed new tables, links, and interfaces to be added incrementally.

This iterative process also helped identify technical issues during development, including inconsistent gene name mappings, the need to remove duplicate records, the requirement to assign unique identifiers across generated CSV files, and the need to validate links between genome contigs, CDS records, transcriptome sequences, and annotation tables. Each module was tested individually before being integrated into the web interface. Feedback from researchers was used to refine search options, result displays, and tool accessibility.

The hybrid approach is therefore presented not as a formal performance comparison against a conventional development model, but as a practical case-study workflow for developing a species-specific bioinformatics database. Its value lies in showing how structured database design can be combined with iterative refinement when biological datasets, user needs, and analytical requirements evolve during development.

### Functional and non-functional requirements

The development of the Fingerroot Genome Database was guided by a set of functional and non-functional requirements identified during the feasibility study and consultation with domain researchers. The main functional requirements were to support the storage, retrieval, and integration of genome sequences, transcriptome sequences, coding sequences, protein annotations, repeat annotations, non-coding RNA annotations, and gene name mappings. The database also needed to provide user-oriented query functions using biologically meaningful identifiers such as contig ID, CDS ID, transcript ID, protein ID, and gene name. In addition, the system was required to integrate sequence analysis and visualization tools, including BLAST-based similarity search and genome browsing, to support exploratory analysis by plant genomics researchers.

The non-functional requirements focused on data integrity, usability, scalability, maintainability, performance, and reliability. Data integrity was essential because the database integrates multiple interrelated biological datasets, where incorrect links between genome, transcriptome, CDS, and annotation records could affect biological interpretation. Usability was addressed through a web-based interface that allows users to access the data without requiring direct database query skills. Scalability was considered in the database schema design so that additional omics layers, updated annotations, or improved genome assemblies can be incorporated in future versions. Maintainability was supported through modular development, database normalization, and clear separation between data storage, query interfaces, and analysis tools. Performance requirements included efficient retrieval of large sequence records and responsive execution of common queries. Reliability was addressed through server-based deployment, versioning, and backup mechanisms to reduce the risk of data loss and support long-term availability.

## Data collection and sources

*Boesenbergia rotunda* is cultivated in regions across India, Indonesia, Thailand, Malaysia, and Southern China (Eng-Chong et al., 2012). The plant holds substantial commercial value owing to its rhizomes and shoots, which are used to flavour food and have widespread application in traditional medicine (Eng-Chong et al., 2012; Jirakiattikul et al., 2021). The rhizomes, with a rich history of traditional use across various cultures, have been employed in traditional medicine to address conditions like cough, muscular pain, rheumatism, gout, infections, inflammatory lesions, and gastrointestinal disorders (Eng-Chong et al., 2012). Extensive research into the secondary metabolites of *B. rotunda*, particularly within its rhizome extracts, has underscored its therapeutic potential. Key flavonoid compounds like panduratin A, 4- hydroxypanduratin, and cardamonin have been reported to exhibit potential biological and pharmacological effects (Taheri et al., 2022), including anti-inflammatory properties (Tuchinda et al., 2002; Rosdianto et al., 2020), anti-tumor activity against various cancers (Break et al., 2021; Kirana et al., 2007; Liu et al., 2018; Tanigaki et al., 2019; Win et al., 2008), and antimicrobial actions against notable pathogens including SARS-CoV-2 in human airway epithelial cells (Kanjanasirirat et al., 2020). The ethnomedicinal significance of *B. rotunda* has ignited interest in exploring cell and tissue cultures for secondary metabolite production (Taheri et al., 2022). However, the current knowledge of the underlying biosynthetic pathways is sparse (Taheri et al., 2022; Boonyarattanasoonthorn et al., 2023).

In this manuscript, genome and transcriptome sequences used to build the database were previously published (Taheri et al., 2022) and are available at https://www.ncbi.nlm.nih.gov/bioproject/PRJNA712941 and the raw sequence data used for genome assembly, mRNA sequencing (RNA-Seq) and whole-genome bisulfite sequencing (BS-Seq) are available at the open science platform at Universiti Malaya at https://doi.org/10.22452/RD/KVOL0C. The original sample material was *Boesenbergia rotunda* (L.) Mansf., Malaysia, Monoisolate. Total genomic DNA was extracted from leaves of *B. rotunda*. Library construction and *de novo* sequencing on the Illumina HiSeq2000 and HiSeq2500 platform (Illumina, San Diego, CA) and the PacBio RS II platform (Pacific Biosciences of California, Inc., United States) were conducted at BGI Shenzhen (Shenzhen, China). Total RNA was isolated from *ex vitro* leaf (EVL), embryogenic callus (EC), dry callus (DC), watery callus (WC), and *in vitro* leaf of regenerated plants (IVL) (n = 3) were used for library construction and sequencing on the Illumina HiSeq2000 platform (Illumina, San Diego, CA) at BGI-Shenzhen (Shenzhen, China).

The *B. Rotunda* scaffold-level genome assembly and associated raw data used to construct the Fingerroot Genome Database were sourced from the NCBI database BioProject PRJNA712941 (Taheri et al., 2022). The transcriptome was assembled following the methodology outlined in Taheri et al. (2022). The tandem repeat and non-coding RNA annotations, along with translated protein-coding genes, the predicted-coding sequence (CDS), and protein annotation data, all outlined in Taheri et al.‘s study, were structured into fifteen distinct Comma Separated Values files (Table 2).

**TABLE 2:** Names of the fifteen Comma Separated Values (CSV) files and their contents.

CSV file name	File content
B_sequences.csv	Translated protein sequences, each assigned with a unique protein ID
ginger.pep.fa.blast.cog.csv	Top five alignment results and annotations of the translated protein sequences with COG database
ginger.pep.fa.blast.kegg.csv	Top five alignment results and annotations of the translated protein sequences with KEGG database
ginger.pep.fa.blast.swissprot.csv	Top five alignment results and annotations of the translated protein sequences with Swissprot database
ginger.pep.fa.blast.trembl.csv	Top five alignment results and annotations of the translated protein sequences with TrEMBL database
ginger.pep.fa.blast.iprscan.csv	Top ten alignment results and annotations of the translated protein sequences with InterPro database with InterProScan tool
annotation.csv	Top annotation data from all five databases mentioned above
coding.csv	Information regarding predicted protein coding sequences
mirna.csv	Annotated miRNA genes
rrna.csv	Annotated rRNA genes
trna.csv	Annotated tRNA genes
snrna.csv	Annotated snRNA genes
trf.csv	Tandem repeats identified with the tandem repeat finder (TRF) (Benson, 1999)
repeatmasker.csv	Repeats identified with Repeat Masker v.4.0.7 (http://www.repeatmasker.org/)
repeatproteinmask.csv	Repeats identified with Protein Repeat Mask v.4.0.7 (http://www.repeatmasker.org/)

### Functional annotation and gene mapping

Gene name mapping was performed to allow users to query with gene names. The process utilized subject accession ID data obtained from protein annotation in which the *in silico* translated gene products were aligned with Swiss-prot (Bairoch and Boeckmann, 1991; Duvaud et al., 2021) and TrEMBL (Bairoch and Apweiler, 1996; Duvaud et al., 2021) databases. Gene name mapping was executed through the utilization of a Bioconductor package named “UniProt.ws” within the R Studio environment. The ginger.pep.fa.blast.swissprot.csv” file was loaded into an R data frame and Swissprot accession IDs were extracted from the “Subject_id” column in this file. The “mapUniProt” function from the UniProt.ws library was then employed to map the Swissprot accession IDs to gene names. The outcome was recorded in a CSV file, encompassing two columns: “Subject_id_swissprot” and “Gene_name”.

To validate the mapping outcome against data retrievable from the UniProt database (The UniProt Consortium, 2023), a selection of random rows from this CSV file was inspected. Subject_id duplicates were removed in the resultant CSV file. This refined CSV file was subsequently labelled as “swissprot_geneName.csv”. Subsequently, each row within the swissprot_geneName.csv was assigned a unique identifier known as the “SP_geneName_ID” to facilitate better integration of the data into the database later in the subsequent step. Parallel procedures were repeated for the TrEMBL blast data stored in the file “ginger.pep.fa.blast.trembl.csv.” The CSV file generated from this process was designated as “trembl_geneName.csv.”, each row of this file was given a unique identifier named “TR_geneName_ID”.

### Data cleaning and normalisation

Certain entries in both the “swissprot_geneName.csv” and “trembl_geneName.csv” files displayed incomplete gene name mapping, a discrepancy attributed to a potential error originating from the UniProt.ws library or the mapUniProt function. For instance, in the case of the protein sequence with the “Subject_id_swissprot” value of “Q9SV13,” it was mapped to two distinct gene names: “1″and “SULTR3.” Subsequent investigation of “Q9SV13″in the UniProt database unveiled that the appropriate gene name was “SULTR3; 1.” Analogous occurrences of such data discrepancies were identified in both CSV files and corrected manually. The specific entries impacted by this error are documented in (Supplementary Table S1), accessible in the appendix section.

### Data integration: linking transcriptome and genome

Linking the assembled genome to transcriptome was required to integrate transcriptome data into the database. This involved obtaining the coding sequence (CDS) from the genome contigs. The process began by converting the coding.csv file into a bed format, which was then used to extract the CDS from the genome contigs (ginger.genome.fa) using BEDtools (Quinlan and Hall, 2010). The resulting CDS were then saved in a FASTA file. Notably, since BEDtools utilize zero-base coordinate system, the start position in the converted bed file was reduced by 1 to ensure accurate extraction of the coding sequence. Subsequently, these CDS query sequences were subjected to a BLAST against the transcriptome contigs which were stored in a FASTA file. The BLAST results were displayed in format 6, including query length and subject length information. Subsequently, the entries with zero gaps in the BLAST results were extracted. From these query sequences, entries with a query length greater than or equal to 303 base pairs (bp) or 100 amino acids were selected and labelled as “Coding Sequence”. Conversely, entries with a query length less than 303 bp or 100 amino acids are classified as “Small Coding Sequence”. Further extraction was performed on each category for entries with zero mismatch, the query start position equals the query length or 1, and the query end position equals the query length or 1. This step isolates entries with full query sequences, regardless of their sense orientation. These entries were then labelled as “full length Coding Sequence” and “full length Small Coding Sequence” respectively.

### Data structuring

To prepare the CDS, transcriptome, BLAST results labelled as “Coding Sequence”, and “Small Coding Sequence” for integration into the database, several steps were taken. Initially, the CDS in FASTA format underwent conversion to CSV format. Subsequently, duplicates within this file were eliminated, and adjustments were made to restore the start positions to their original values (increased by 1). The resulting CSV file now includes the FASTA header (designated as gene position in the database), encompassing contig ID, start and end positions, and the CDS itself. The extracted start position, end position, and contig ID were organized into separate columns for each entry, and a unique CDS ID was assigned to each of these entries.

The FASTA file containing the transcriptome contigs was converted to CSV format, resulting in a file named “Transcript_sequence.csv” with two columns: the FASTA header (referred to as “Transcript_header” in the database) and transcriptome contig sequences (referred to as “Transcript_sequence” in the database). Each entry was assigned a unique transcript ID. Likewise, the FASTA file containing genome contigs was converted into a CSV file named “Genome_contig.csv,” where each sequence was assigned a unique contig ID.

Finally, to prepare the BLAST results, a CSV file was generated to store the FASTA headers (position) of the coding sequence and the transcript ID(s) of their corresponding matched transcript(s) for the data labelled as “Coding Sequence”. Each entry was assigned a unique ID. The same procedure was followed for the data labelled as “Small Coding Sequences”, “full-length Coding Sequences”, and “full-length Small Coding Sequences”.

### Database design using the entity relationship modeling

**FIGURE 1:**
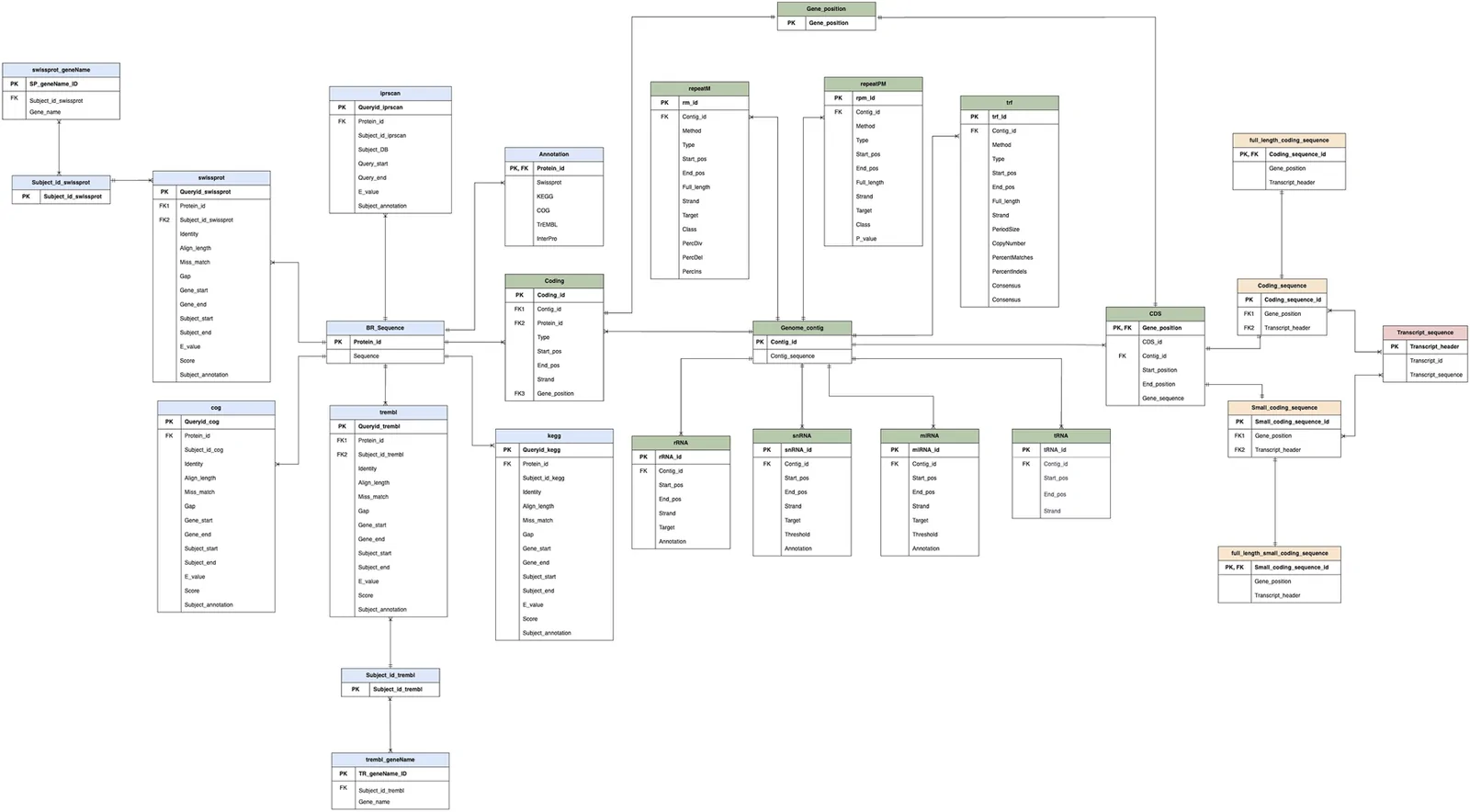
The entity-relation (ER) diagram of the fingerroot database.

All collected and processed data were structured into twenty-seven relational tables, collectively constituting the database’s relational architecture (Figure 1). The fifteen original CSV files (Table 2), along with the twelve subsequently created CSV files (Table 3), were individually converted into distinct tables within the database. The database can be broadly segmented into four distinct yet interconnected datasets. The ER diagram (Figure 1) illustrates these datasets, with each color symbolizing a different dataset. The “BR_Sequence” table houses translated protein sequences linked to their annotations from five different databases, gene name mapping information, and the coding table. Serving as a pivotal connection, the coding table links to the “Genome_contig” table, which contains genome contig sequences. The “Genome_contig” table is further linked to other tables containing repeat annotation data and non-coding RNA annotation data. It is also connected to the transcriptome through the “CDS” table, and two additional tables named “Coding_sequence” and “Small_coding_sequence,” both containing sorted BLAST entries. The tables “full_length_coding_sequence” and “full_length_small_coding_sequence” are associated with the “Coding_sequence” and “Small_coding_sequence” tables, respectively, facilitating an interface function for discerning the full-length status of the query sequence in these tables.

**TABLE 3:** Names of the twelve Comma Separated Values (CSV) files and their contents.

CSV file name	File content
Genome_contig.csv	Genome contigs, each assigned with a unique contig ID
Subject_id_swissprot.csv	Cleaned subject IDs extracted from ginger.pep.fa.blast.swissprot.csv (Table 2)
swissprot_geneName.csv	Subject IDs from Subject_id_swissprot.csv and their corresponding gene name(s) from Swissprot database
Subject_id_trembl.csv	Cleaned subject IDs extracted from ginger.pep.fa.blast.trembl.csv (Table 2)
trembl_geneName.csv	Subject IDs from Subject_id_trembl.csv and their corresponding gene name(s) from TrEMBL database
Gene_position.csv	Position of coding sequences in the genome contigs. This csv file was then transformed into the “Gene_position” table to connect the “Coding” table and the “CDS” table in the database (Figure 1)
CDS.csv	Coding sequences extracted from the genome contigs, each coding sequence was assigned with a unique CDS ID
Transcript_sequence.csv	Transcriptome contigs, each has a unique transcript (FASTA) header and was also assigned a unique transcript ID
Coding_sequence.csv	BLAST entries labelled as “Coding Sequence”, each was assigned a unique Coding sequence ID
Small_coding_sequence.csv	BLAST entries labelled as “Small Coding Sequence”, each was assigned a unique Small coding sequence ID
full_length_coding_sequence.csv	“Coding Sequence” BLAST entries with full query sequences - which were labelled as “full length Coding Sequence”
Full_length_small_coding_sequence.csv	“Small Coding Sequence” BLAST entries with full query sequences - which were labelled as “full length Small Coding Sequence”

### Agile based iterative development

Data storage and management were implemented using MySQL (https://dev.mysql.com). Data from CSV files obtained in previous steps were imported directly into the MySQL database through SQL commands. Web user interfaces to access and explore the datasets stored in the database were created utilizing the LAMP (Linux, Apache, MySQL, and Python) stack, HTML (HyperText Markup Language), CSS (Cascading Style Sheets), and JavaScript.

Third-party software, NCBI BLAST+ (Camacho et al., 2009), and SequenceServer (Priyam et al., 2019), were employed for the secondary development of the sequence alignment tool integrated into the database. To enhance user interaction and analysis of BLAST results, SequenceServer (version 3.1.0) was chosen as the tool to execute BLAST queries. Following its download and installation, adhering to instructions outlined on https://sequenceserver.com/doc/, the software was configured and set to initiate automatically upon the server’s start up. Its interface was then integrated into an HTML webpage, enabling users to access SequenceServer’s functionality directly from the database web portal. The BLAST + software (2.14.0+) was downloaded automatically by SequenceServer once it was installed and configured. Four distinct databases were established, each derived from the genome, transcriptome, CDS, and protein sequences provided in FASTA format.

In addition to that, JBrowser2 (Diesh et al., 2023) was integrated into the database to serve as tool for genome visualization. JBrowse and JBrowse2 were installed and set up following the instructions on https://jbrowse.org/jb2/docs/quickstart_web/. The complete *B. rotunda* genome assembly was added to the software, and the GFF file converted from coding.csv was included as a track in the browser.

### Architectural decisions and justification

The Fingerroot Genome Database was designed using a modular three-layer architecture consisting of a data layer, application/interface layer, and integrated analysis layer. The data layer was implemented using a relational database model to organize genome, transcriptome, CDS, annotation, and mapping data into structured tables. This architectural decision was appropriate because the project involved multiple highly related datasets that required clear primary-key and foreign-key relationships. A relational schema also supported normalization, reduced redundancy, and enabled consistent linking between sequence records and their associated annotations.

The application/interface layer was developed as a web-based system to improve accessibility for researchers. A web interface was selected because it allows users to search and explore the database without installing specialized software or directly interacting with SQL commands. This was particularly important because the target users include biologists and plant genomics researchers who may have different levels of computational expertise. The interface was developed iteratively so that search pages, result pages, and sequence display functions could be refined based on feedback from researchers.

The integrated analysis layer included BLAST functionality through SequenceServer and genome visualization through JBrowse2. This decision was made to move the database beyond passive data storage and provide basic analytical functions within the same platform. BLAST integration enables users to compare query sequences against the genome, transcriptome, CDS, and protein datasets, while JBrowse2 allows interactive exploration of genomic regions and annotation tracks. The use of established third-party bioinformatics tools reduced development complexity, improved reliability, and allowed the system to provide familiar analysis functions to the bioinformatics community.

The architecture therefore reflects a balance between structured database design and flexible, user-driven exploration. The use of a relational backend ensures data consistency, while the modular interface and integrated tools support extensibility and future enhancement. This approach is consistent with the hybrid methodology proposed in this manuscript, where the Database Development Life Cycle provides structure and Agile principles support iterative improvement.

### Choice of technologies and technical challenges

MySQL was selected as the backend database management system because it is widely used, open source, stable, and suitable for relational data management. Its support for indexing, structured queries, and table relationships made it appropriate for storing and linking the 27 tables that form the Fingerroot Genome Database. The LAMP stack was selected for web deployment because it provides a mature and cost-effective environment for hosting database-driven biological web resources. HTML, CSS, and JavaScript were used to develop the user interface because they are standard web technologies that support browser-based access and interactive presentation of search results.

NCBI BLAST+ was selected because it is a standard tool for sequence similarity searches in bioinformatics. SequenceServer was used to provide a user-friendly graphical interface for BLAST execution and result interpretation. This allowed BLAST functionality to be integrated into the database portal without building a sequence alignment interface from the beginning. JBrowse2 was selected as the genome browser because it supports interactive visualization of genome assemblies and annotation tracks, making it suitable for exploring coding regions and genomic features in B. rotunda.

Several technical challenges were encountered during development. First, the integration of heterogeneous datasets required careful data cleaning, normalization, and restructuring because genome sequences, transcriptome assemblies, CDS records, protein annotations, repeat annotations, and gene name mappings were originally available in different formats. Second, linking genome and transcriptome data required additional BLAST-based processing and classification of coding sequences, including the identification of coding sequences, small coding sequences, and full-length matches. Third, gene name mapping required manual validation and correction because some mappings from UniProt-derived data contained incomplete or inconsistent gene names. Fourth, the conversion of sequence files and annotation data into database-ready CSV tables required careful assignment of unique identifiers to preserve relationships across tables. Fifth, integrating external tools such as SequenceServer and JBrowse2 required configuration, server deployment, and testing to ensure that users could move smoothly between database query results, BLAST searches, and genome visualization.

These challenges were addressed through iterative development, unit testing, integration testing, manual validation of selected records, and feedback from domain researchers. The Agile component of the hybrid methodology was particularly useful because it allowed technical issues and new requirements to be addressed progressively without redesigning the entire database structure.

## Results

### Database contents

The Fingerroot Genome Database is available at/brotunda.um.edu.my and encompasses a comprehensive repository of genetic information, featuring a total of 10,627 assembled genome contig sequences and 95,847 assembled transcriptome sequences for *B. rotunda*. Each genome contig is uniquely labelled with a contig ID, while each transcriptome sequence is assigned a distinct transcript ID. It also houses 65,535 sequences annotated as tandem repeats, along with Transposons and Transposable Elements (TEs) identified using Repeat Masker and Protein Repeat Mask. Annotated information on non-coding RNA is also provided, including details on 213 microRNA (miRNA), 3173 transfer RNA (tRNA), 486 ribosomal RNA (rRNA), and 2136 small nuclear RNA (snRNA) genes.

Additionally, the Fingerroot database provides information on the 73,102 CDSs, along with its corresponding translated protein sequence. The database also hosts protein annotations sourced from five databases which are COG, InterPro, KEGG, Swissprot, and TrEMBL. These proteins are also mapped to Swissprot and TrEMBL gene names to facilitate more user-friendly query. The integration of transcriptome and genome data is achieved through internal linking via BLAST results. By sorting these results, the 55,939 query sequences (CDSs) in the BLAST entries with zero gaps are further classified into 55,361 Coding Sequence (with 69 of them being full-length Coding Sequence) and 578 Small Coding Sequence (with 21 of them being full-length Small Coding Sequence) facilitating efficient identification of coding gene to meet various research needs.

### Tools for database exploration

To facilitate analysis within the Fingerroot Genome Database, various tools, including BLAST+ and JBrowse2 are currently available. The database executes BLAST + through SequenceServer, allowing users to initiate searches across four different databases. This process aids in result retrieval and assessment by cross-referencing contig IDs, transcript IDs, CDS IDs, and protein IDs within the database. The integration of JBrowser2 (Diesh et al., 2023) enhances genome visualization capabilities within the database framework. These tools offer distinct features for navigating, analyzing, and interpreting genomic data. Together, they contribute to a comprehensive and interactive exploration of the data stored in the database.

### Use case: flavonoid pathway exploration

As previously mentioned, flavonoids are among the most valuable secondary metabolites extracted from *B. rotunda* herbs. The biosynthesis of flavonoids has been a longstanding focus of intense research in plant biology. Flavonoids are produced through the phenylpropanoid pathway, initiating with the conversion of phenylalanine to cinnamic acid by the enzyme phenylalanine ammonia-lyase (PAL) (Liu et al., 2021). To further investigate the flavonoid biosynthesis pathway in *B. rotunda* herbs, it is essential to identify the underlying genes associated with the pathway and explore the transcripts linked to these genes within this specific plant. The Fingerroot Genome Database can be employed for deciphering the genetic components of the flavonoid biosynthesis pathway in these herbs. We utilize the enzyme phenylalanine ammonia-lyase (PAL) in the phenylpropanoid pathway as a use case of the Fingerroot Genome Database presented in this paper.

**FIGURE 2:**
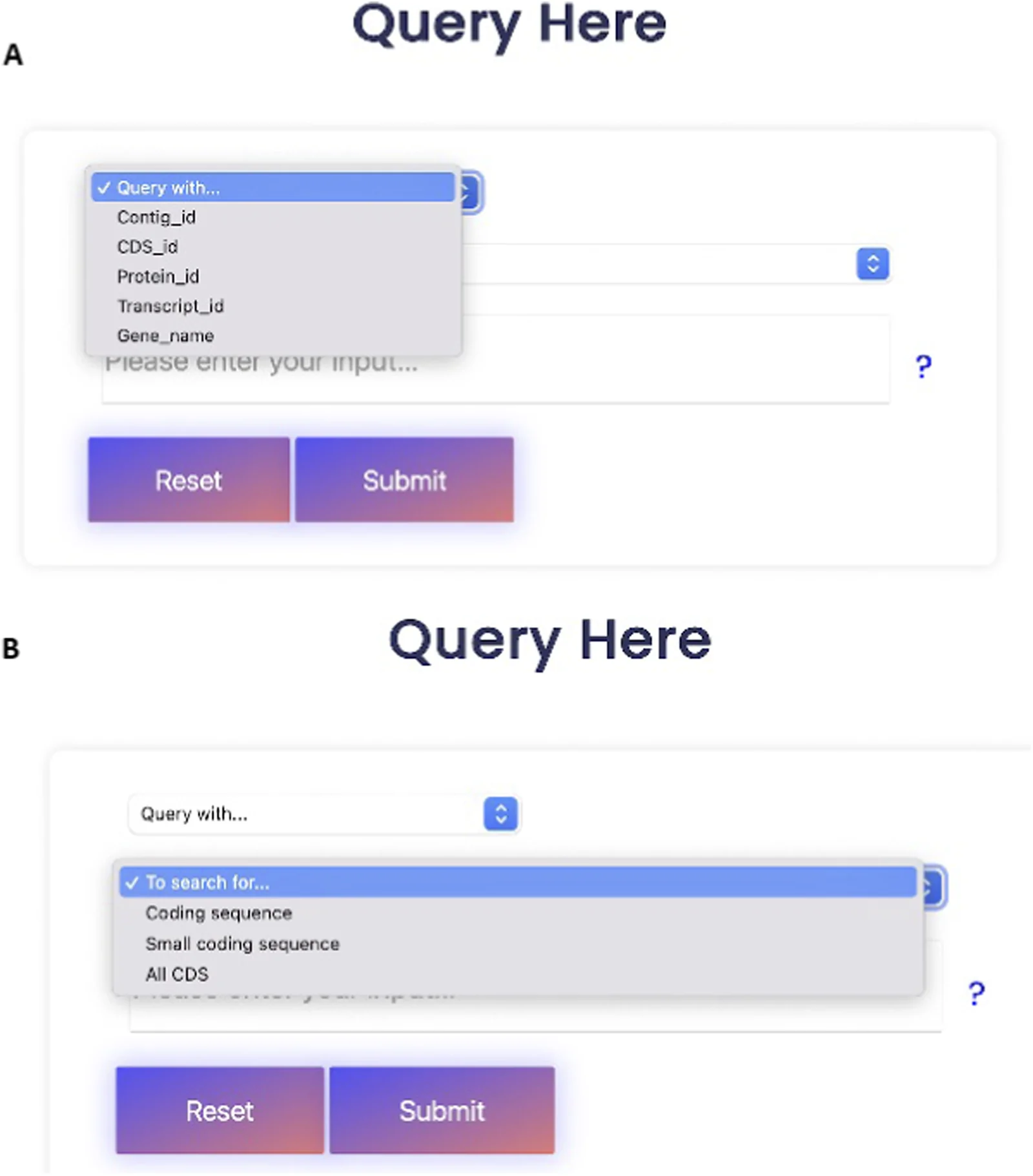
Query options available for users. **(A)** Users can choose to query with contig ID, CDS ID, protein ID, transcript ID, or gene name. **(B)** To search for coding sequence (sequence longer than or equal to 303bp or 100 amino acids), small coding sequence (sequence shorter than 303bp or shorter than 100 amino acids), or all CDS (regardless of sequence length).

**FIGURE 3:**
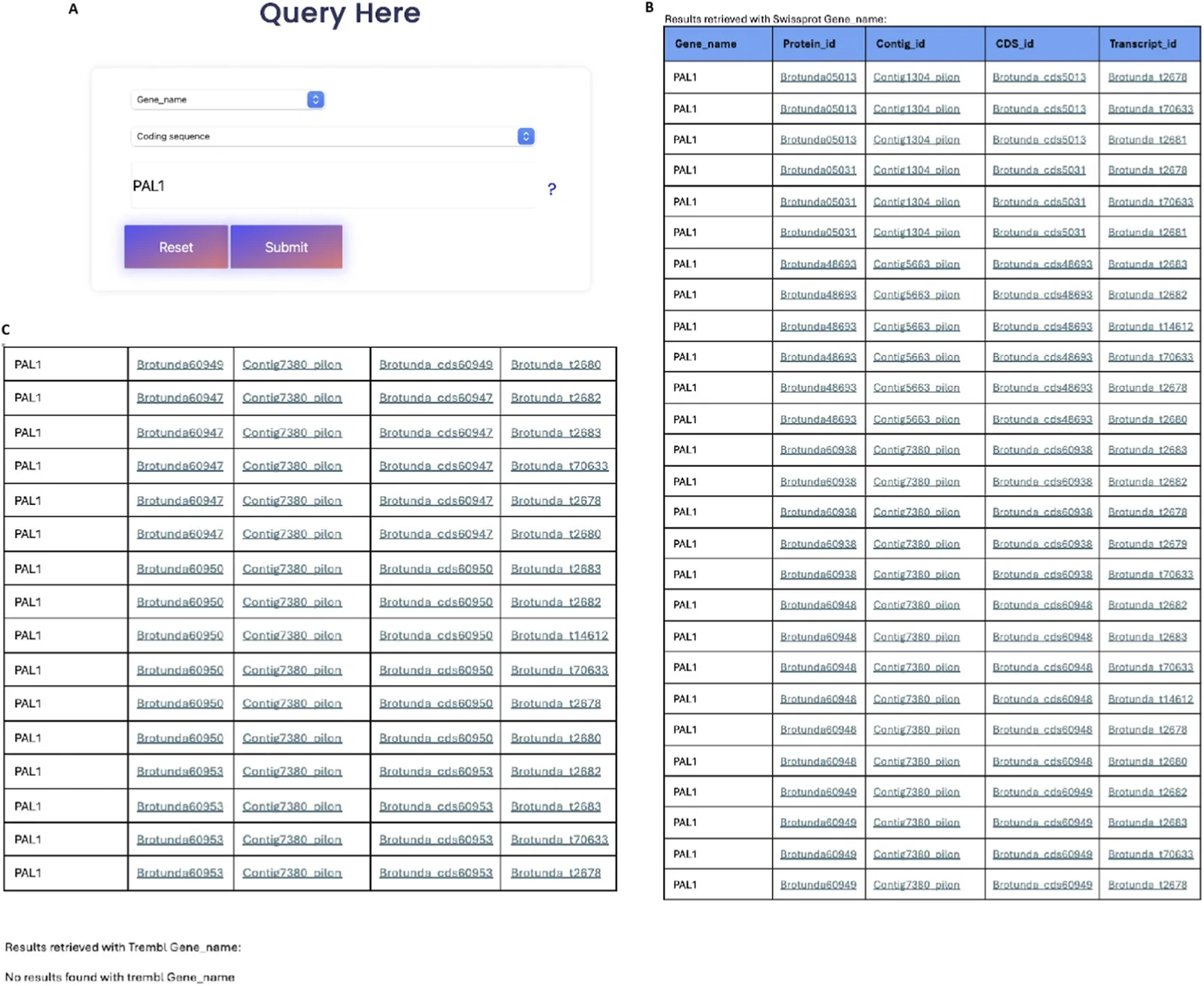
Fingerroot genome database query. **(A)** Search for coding sequence with gene name “PAL1”. **(B-C)** Query result for gene name “PAL1”.

The query service of the database enables inquiries using five distinct parameters: contig ID, CDS ID, transcript ID, protein ID, and gene name (Figure 2). Users are then required to narrow down their queries to specific categories, including “Coding Sequence”, “Small Coding Sequence”, or they can select the “All CDS” option (Figure 3) when uncertainty arises about the sequence’s length. Once this choice is made, users will then need to provide the necessary query input to initiate the search process.

**FIGURE 4:**
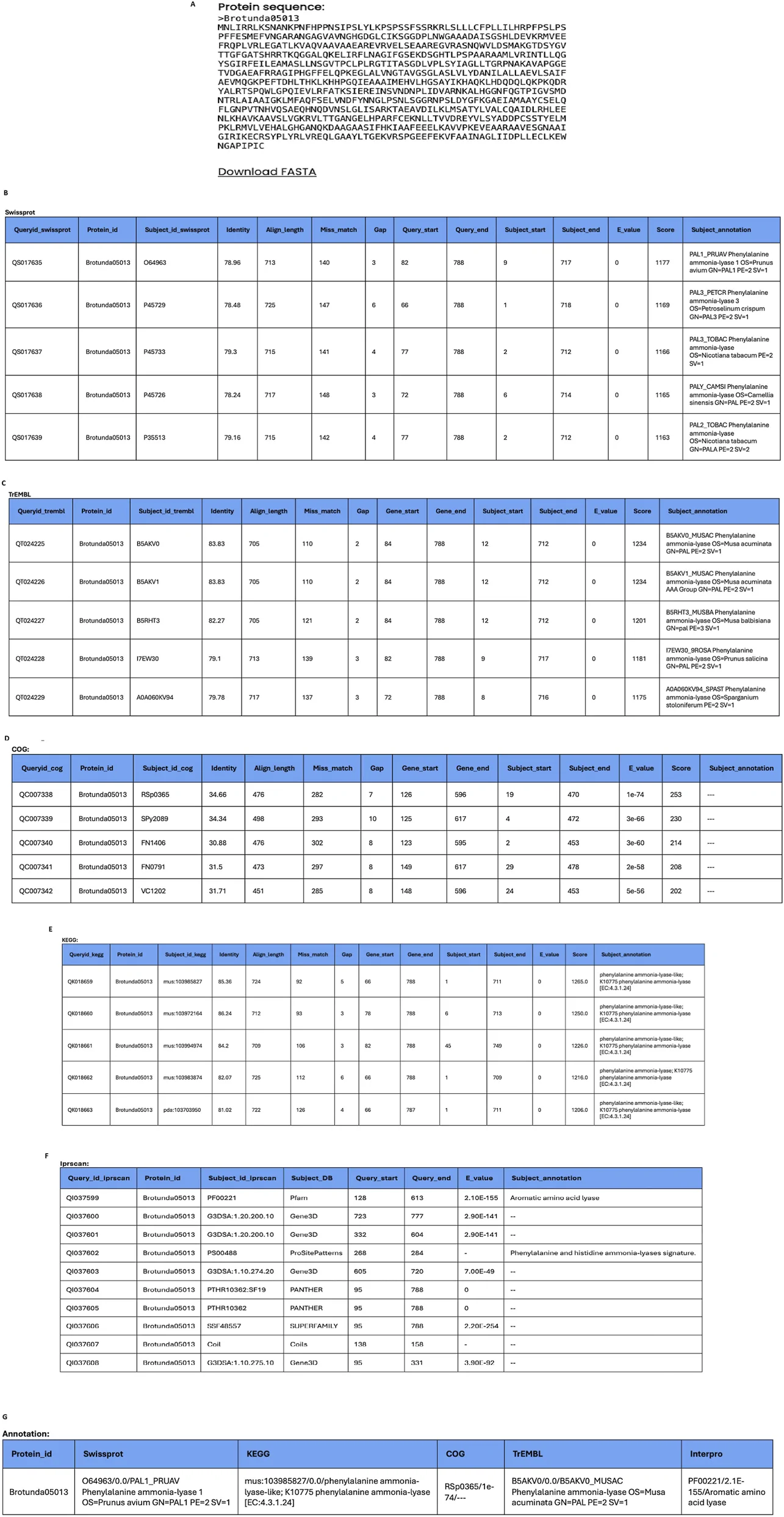
In silico-translated protein sequence with the protein ID “Brotunda05013” from query results, and its annotations. **(A)** Protein sequence in FASTA format. **(B)** Top five alignment results and annotations from the Swissprot database. **(C)** Top five alignment results and annotations from the TrEMBL database. **(D)** Top five alignment results and annotations from the COG database. **(E)** Top five alignment results and annotations from the KEGG database. **(F)** Top ten alignment results and annotations from the InterPro database. **(G)** Combined top alignment results and annotations of the same protein sequence from all five databases.

**FIGURE 5:**
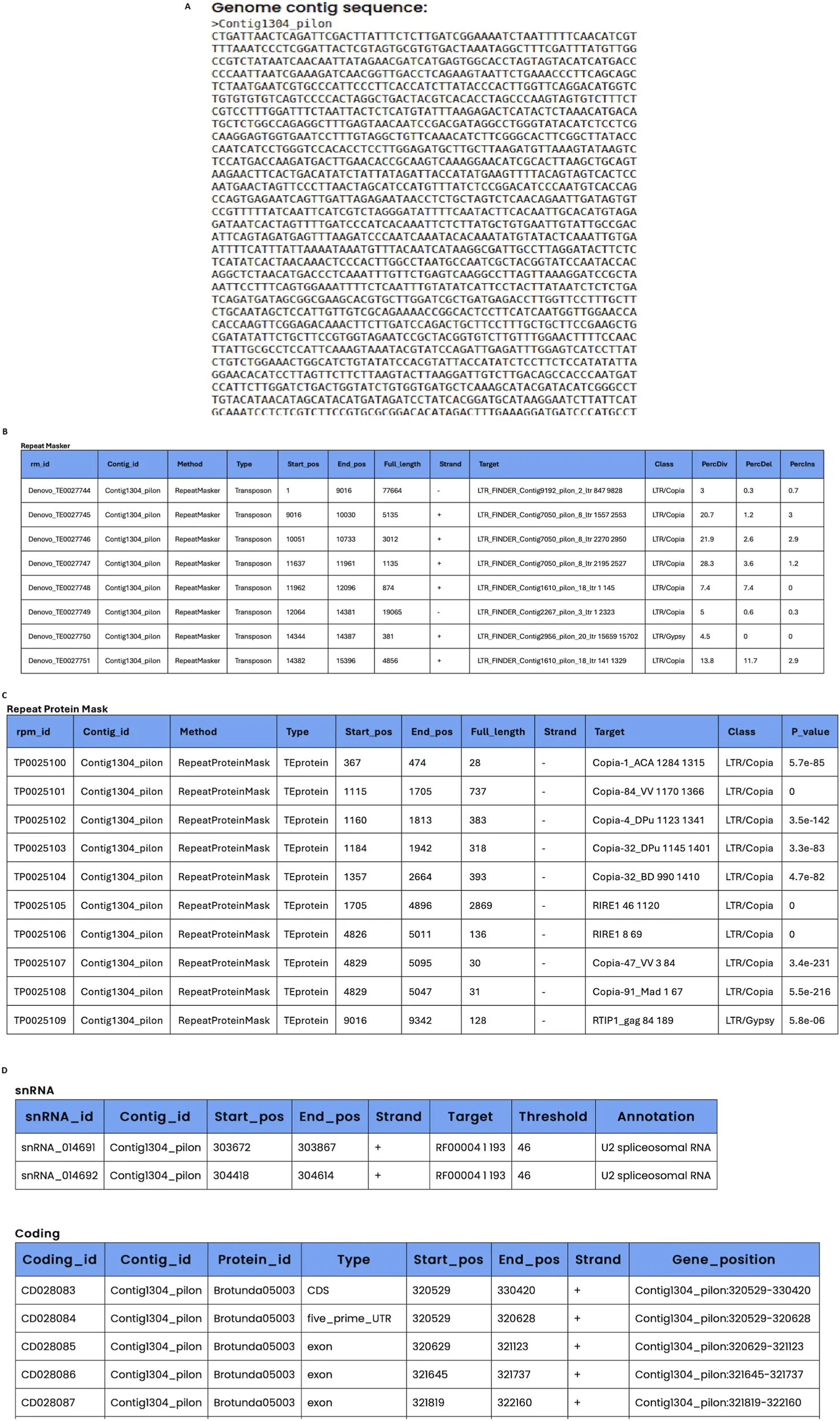
Genome contig with contig ID “Contig1304_pilon” from the query results, and its annotations. **(A)** Genome contig sequence in FASTA format. **(B)** Identified repeats using Repeat Masker. **(C)** Repeats identified with Protein Repeat Mask. **(D)** Annotations for the snRNA genes and coding sequence in the genome contig.

**FIGURE 6:**
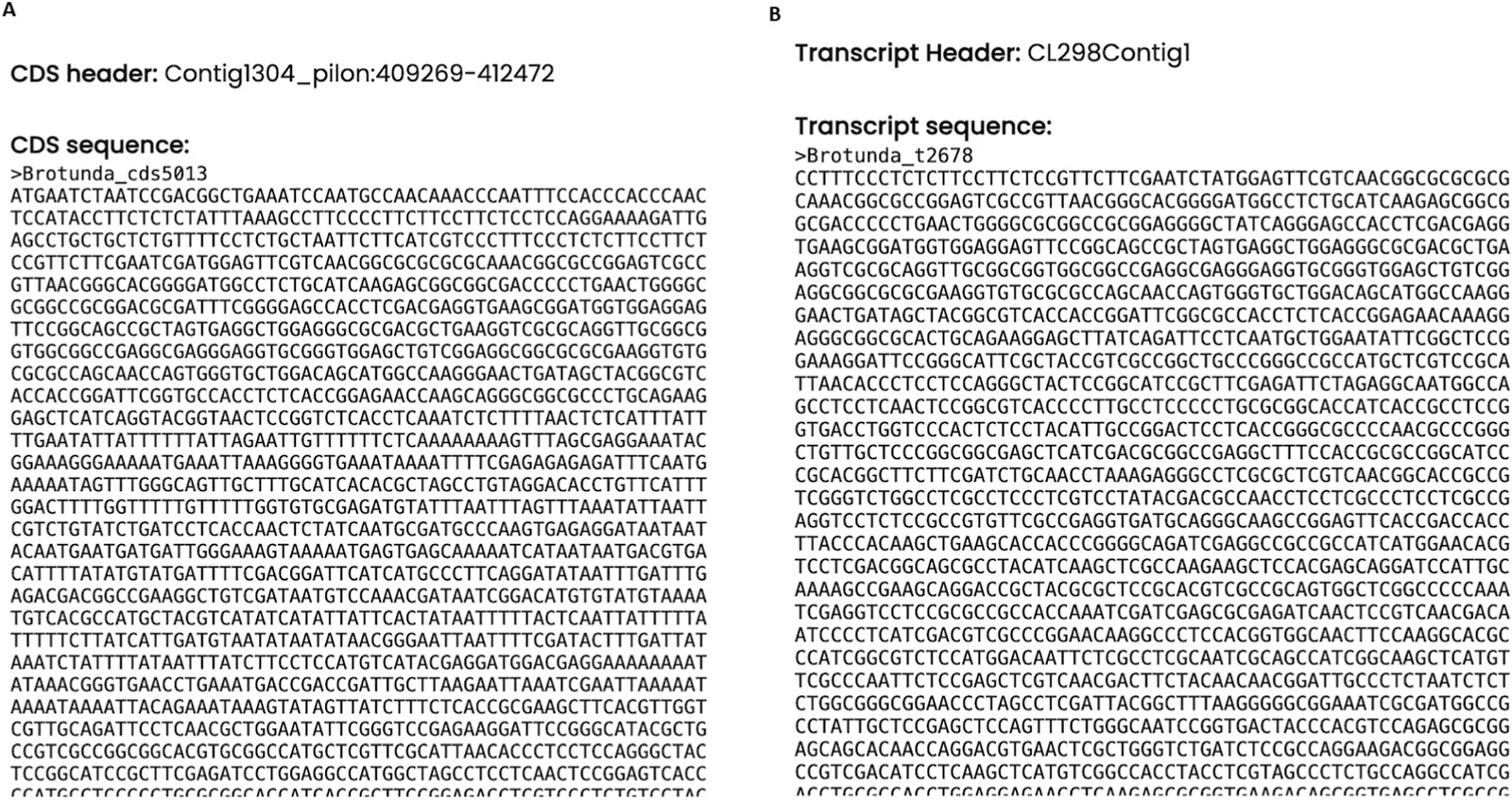
CDS and transcript sequence respective to gene name “PAL1” and protein sequence “Brotunda05013” according to the query results. **(A)** CDS header (position) and sequence in FASTA format for CDS with CDS ID “Brotunda_cds5013”. **(B)** Transcript header and sequence in FASTA format for transcript with ID “Brotunda_t2678”.

As an example, we will use the gene name of the PAL1 protein from *Prunus avium* (with UniProt accession ID O64963) to query the database and identify related genetic components in *B. rotunda*. Given that this protein comprises 717 amino acids, we will opt for the “Coding Sequence” option in the “To search for” query section (refer to Figure 3A). The query result (Figure 3B,C) provides the users with the translated protein sequences of *B. rotunda* that are associated with the gene name “PAL1,” along with their corresponding CDS IDs, linked transcript sequences, and the genomic contigs where these transcripts and CDS are located. When users interact with the protein ID, such as “Brotunda05013,” on the query result page (Figure 3B,C), they can access the protein sequences in FASTA format (Figure 4A) and explore the annotations for each protein (Figure 4B-F). Likewise, comprehensive details, including the genome contig sequence (Figure 5A), repeat annotations (Figure 5B,C), coding and non-coding RNA annotations (Figure 5D), become accessible by clicking on the contig ID. An illustrative instance is showcased in Figure 5A-D with the genome contig bearing the contig ID “Contig1304_pilon”, as found on the query result page (Figure 3B). Additionally, CDS and transcript sequences are also available in FASTA format through the interaction with CDS IDs and transcript IDs (Figure 3B-E). In this scenario, we chose to demonstrate the functionality by clicking on the CDS ID “Brotunda_cds5013,” corresponding to the protein sequence with the protein ID “Brotunda05013,” and selecting the transcript with the transcript ID “Brotunda_t2678” that was matched with the CDS. The outputs are shown in Figure 6A,B.

**FIGURE 7:**
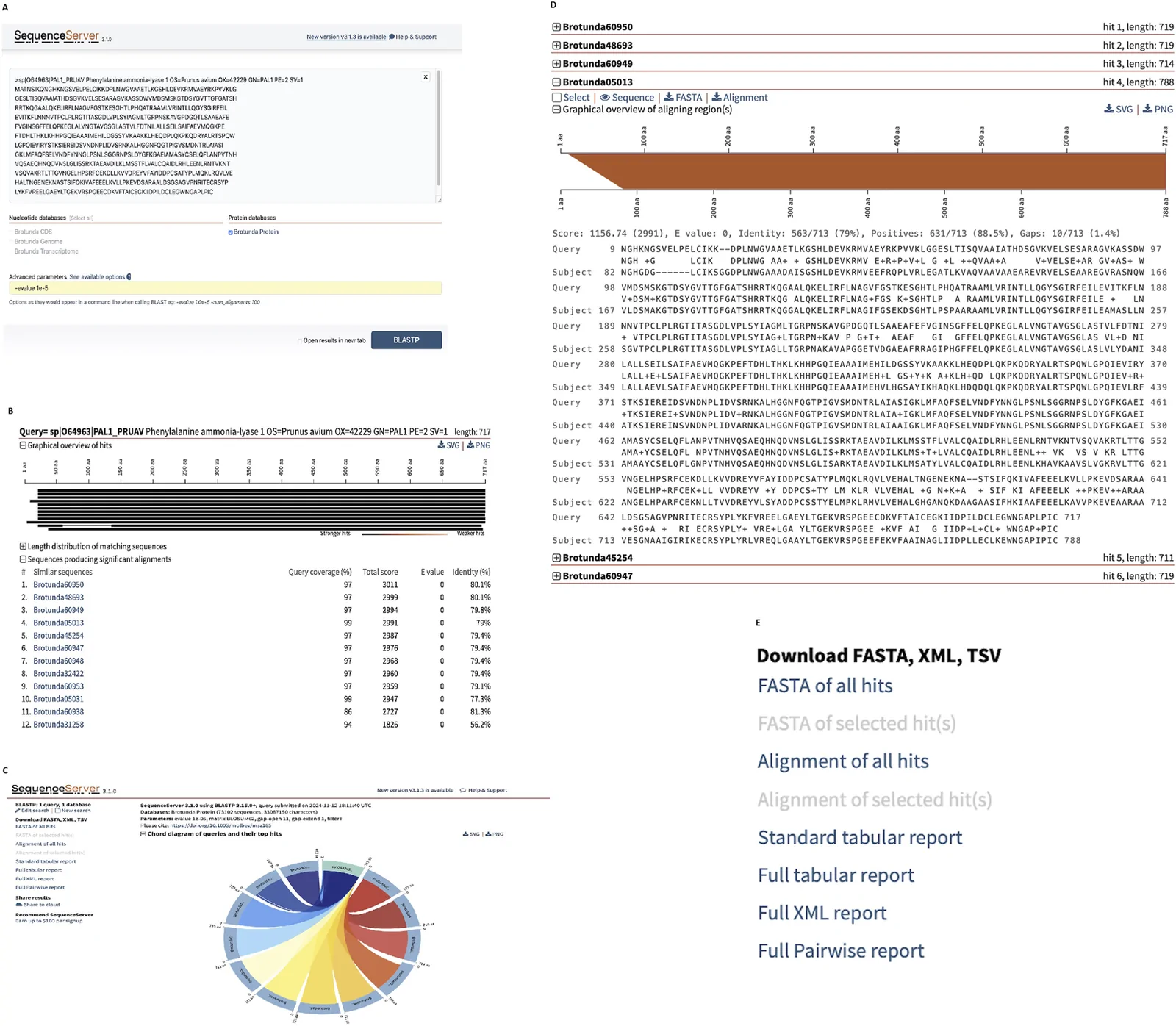
The BLAST query is executed through Sequenceserver in the database. **(A)** Sequenceserver interface when queried with the PAL1 protein sequence and subjected to BLAST against the B. Rotunda protein database. **(B)** The BLAST result displays 12 hits for the query sequence. **(C)** A chord diagram provides a visual representation of the query and its top hits from the BLAST. **(D)** A graphical overview illustrates aligned regions for the protein sequence Brotunda05013. **(E)** Various options are available for downloading FASTA files of matched subject sequences and the BLAST report in different formats.

Different tools integrated into the database can also be utilized to navigate and explore the data associated with PAL1 protein in the database. The PAL1 protein sequence, obtained from UniProt, was aligned to the *B. rotunda* protein database using the “BLAST” tool (Figure 7A). The results reveal 12 hits for the query sequence (Figure 7B), encompassing the 9 protein sequences previously identified through the gene name query, along with 3 additional *B. rotunda* protein sequences (Brotunda45254, Brotunda32422, and Brotunda31258) which were previously unassociated with the gene name “PAL1″ in the database. The BLAST search identified 12 protein hits, including the 9 sequences retrieved through the gene-name-based PAL1 query and 3 additional sequences, Brotunda45254, Brotunda32422, and Brotunda31258, that were not retrieved by the gene-name search. In this PAL1 example, the gene-name query therefore recovered 9 of the 12 BLAST-supported candidates, indicating that gene-name-based searching may under-recall sequences when functional labels are incomplete, inconsistent, or absent in the Swissprot/TrEMBL-derived mapping index. These additional BLAST hits should therefore be interpreted not only as candidates for further biological investigation, but also as evidence that BLAST-based sequence search remains necessary to complement gene-name-based querying.

To analyze the BLAST results, Sequenceserver provides a graphical overview of the hits (Figure 7B), allowing users to explore the query sequence and its top hits through a chord diagram visualization (Figure 7C). Additionally, a graphical overview of the aligning region(s) for each pair of sequences with significant alignments is generated for further inspection (Figure 7D). Within the result page, users are given the option to download all graphical diagrams in either SVG or PNG format. They can choose to download the alignment, as well as the corresponding matched *B. rotunda* protein subject sequence, in FASTA format. This flexibility extends to downloading FASTA files for all or selected hits, alignment files for all or selected hits, and the BLAST report, all available in various formats (Figure 7E).

**FIGURE 8:**
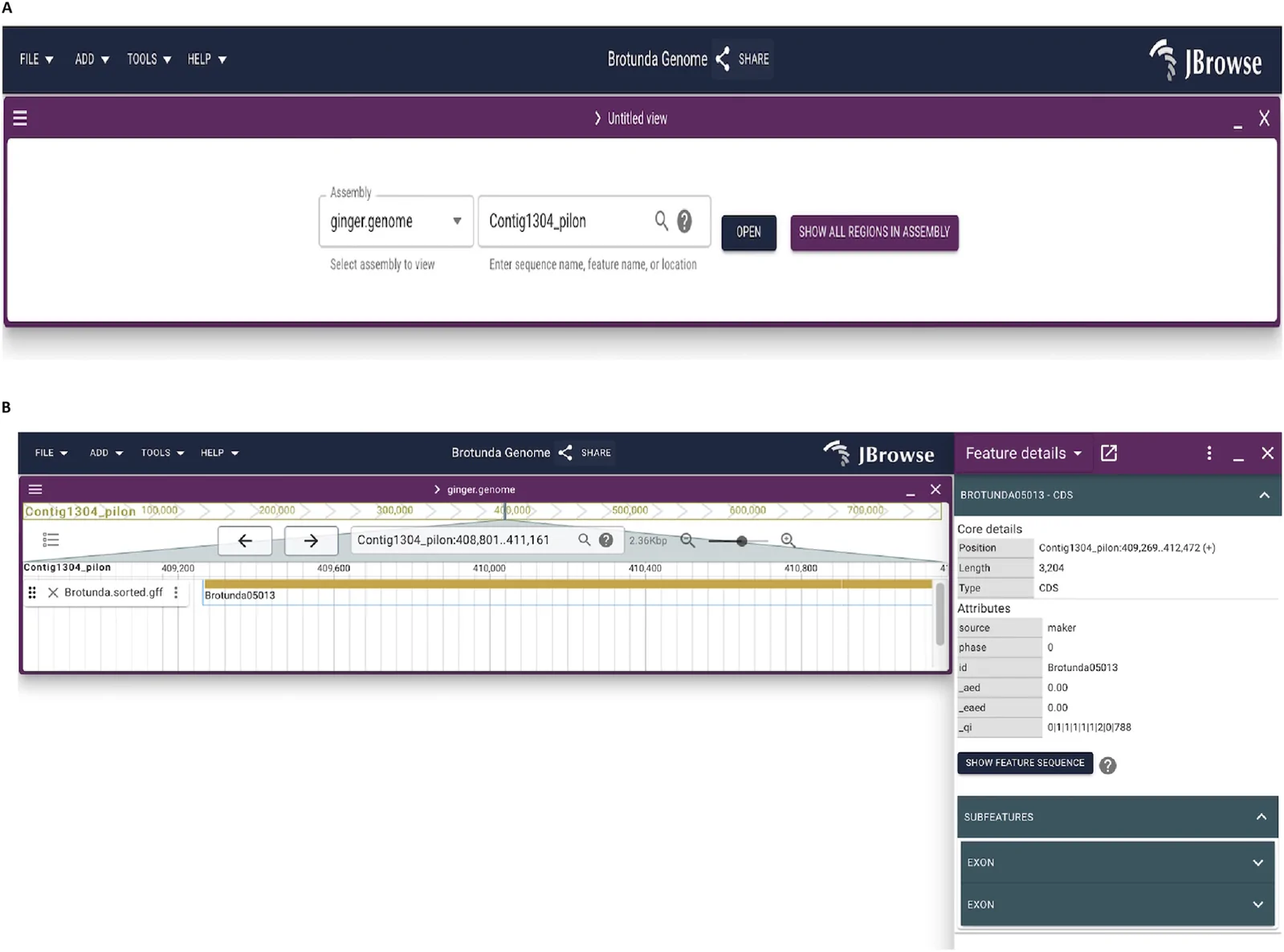
Accessing genomic data through the JBrowse tool in the database. **(A)** Initiating a search for the CDS of the protein sequence Brotunda05013 with the CDS header (Contig1304pilon:409269-412472), previously obtained from gene name query. **(B)** Visualizing the CDS within the JBrowse interface.

Beyond the BLAST tool, users can also leverage JBrowse as an interactive and visual platform to navigate through the genomic landscape of *B. rotunda*. Using protein sequence “Brotunda05013″located at position 409,269-412472 on Contig1304_pilon (Figure 8A), as an example, Figure 8B illustrates how users can visually explore the region of interest.

## Discussion

*Boesenbergia rotunda*, commonly known as Fingerroot ginger, holds immense value due to its commercial and medicinal significance. The completion of the *B. rotunda* genome sequence and deep transcriptome assembly has paved the way for unprecedented insights into the metabolic pathways of its therapeutic compounds. This paper presents the Fingerroot Genome Database, hosting 10,627 genome contig sequences and 95,847 transcriptome sequences, functions as a pivotal repository. Researchers can explore tandem repeats, non-coding RNA annotations, predicted protein-coding sequences, and translated protein sequences with annotations. The database also offers tools including BLAST and JBrowse, facilitating searches, result retrieval, and enhanced genome visualization.

The hybrid methodology presented in this paper ensures database robustness, flexibility, and biological relevance. By integrating agile cycles within Database Development Life Cycle (DDLC), we managed dynamic requirements such as transcriptome assembly and new annotation integration. Compared to traditional systems, this approach minimizes downtime, increases user feedback responsiveness, and scales to future omics layers like proteomics and metabolomics.

Before the establishment of the Fingerroot Genome Database, studies on *B. rotunda* encountered challenges with fragmented data, as valuable information like protein annotations was dispersed across various databases such as KEGG, Swissprot, and COG. By consolidating data from five databases, namely, COG, InterPro, KEGG, Swissprot, and TrEMBL, the Fingerroot Genome Database effectively addresses the issue of scattered data. This centralized platform, equipped with simple tools to meet basic research needs, not only enhances accessibility to genetic materials and information associated with *B. rotunda* but also facilitates the integration of genome and transcriptome data. It establishes a robust foundation for exploring the genetic landscape, gene regulation, and molecular mechanisms underlying various biological processes in *B. rotunda*. In its early stage, the Fingerroot Genome Database actively seeks user feedback and updated information to ensure continuous enhancement and user-friendliness. The database encourages users to report any encountered errors or malfunctions and is open to the possibility of accepting data submissions related to *B. rotunda* or genome assemblies with higher resolution.

The PAL1 use case also highlights an important limitation of the gene-name-based query function. Although gene-name search improves usability by allowing users to search using familiar biological terms, its recall depends on the completeness and consistency of the Swissprot/TrEMBL accession-to-gene-name mapping used to construct the query index. In the PAL1 example, the gene-name query retrieved 9 sequences, whereas direct BLAST search retrieved 12 hits. The three additional BLAST hits were not indexed under the PAL1 gene name, most likely because their corresponding annotations or accession mappings did not contain the expected gene-name label. This demonstrates that gene-name queries may under-recall biologically relevant sequences, particularly for underrepresented plant species where annotations are transferred from homologous proteins and may be incomplete. Therefore, the gene-name search function should be viewed as a user-friendly entry point rather than a replacement for sequence-based search. For pathway exploration or candidate gene discovery, users are encouraged to combine gene-name queries with BLAST searches to improve recall. Future versions of the database will include systematic evaluation of gene-name query recall across selected gene families and pathways, as well as improved synonym handling, annotation updates, and cross-reference expansion to reduce missed candidates.

A current limitation of the database is that the recall of gene-name-based queries has not yet been systematically benchmarked against sequence-based BLAST searches across multiple genes or pathways. The PAL1 case study suggests that gene-name queries may miss some biologically relevant candidates when gene-name mappings are incomplete or inconsistent. Future work will include broader validation of gene-name query performance and the incorporation of expanded synonym dictionaries, updated UniProt mappings, and additional functional annotation sources.

In the future, we plan to continue to work with multomics data integration into the Fingerroot genome database with the aim of this becoming a reference database for ginger plant researchers to address critical challenges in ginger genomic studies. Future efforts will include further development of browser providing enhanced query options expanding analysis capabilities. This database will be improved and updated continuously with new features, improvements to genome annotation, genomic sequences, and availability of other omics data.

The completion of the first complete *B. rotunda* genome sequence through a hybrid assembly strategy, aimed to unlock insights and provide useful information towards understanding the metabolic pathways of the plant’s natural products (Taheri et al., 2022). In addition to that, a deep transcriptome (RNA-seq data) assembly from five *B. rotunda* samples, including various types of callus cultures, and leaves was also carried out in the same study, to provide transcriptome data that allows for the identification of gene expression profiles (Taheri et al., 2022). The availability of the complete sequence data and genomic information in the study highlights the need for a dedicated repository capable of efficiently storing and leveraging them for future research. This database serves as a platform that ensures public accessibility and facilitates dissemination of the available data to researchers and research groups worldwide.

## Conclusion

This study presents the Fingerroot Genome Database as a case study in the development of a species-specific bioinformatics resource for an underrepresented medicinal plant. Rather than serving as a formal validation of one software development methodology over another, the work demonstrates how structured Database Development Life Cycle (DDLC) phases can be combined with Agile-inspired iterative refinement to manage evolving biological data, changing user requirements, and the integration of external analysis tools. The resulting workflow may serve as a practical reference for researchers developing similar custom bioinformatics databases, particularly where heterogeneous omics datasets, functional annotations, and user-facing analytical tools need to be integrated within a single platform.

To the best of our knowledge, the Fingerroot Genome Database is the first dedicated genome database developed specifically for Boesenbergia rotunda. Compared with established multi-species plant genome databases listed in Table 1, this database is narrower in taxonomic scope but more focused in its species-specific integration of B. rotunda genome sequences, transcriptome sequences, coding sequences, functional annotations, gene name mappings, BLAST-based sequence search, and genome visualization. Its novelty therefore lies not in replacing broader comparative plant genomics platforms, but in addressing the absence of a dedicated genomic resource for B. rotunda.

By consolidating available genomic and transcriptomic resources within a single searchable and visual platform, the Fingerroot Genome Database provides a useful resource for researchers interested in the genomics, functional biology, and medicinally important secondary metabolite pathways of B. rotunda. This dedicated focus may support future studies on gene function, pathway exploration, comparative analysis within related Zingiberaceae species, and the continued development of bioinformatics resources for underrepresented medicinal plants.

## Data Availability

Publicly available datasets were analyzed in this study. This data can be found here: https://doi.org/10.22452/RD/KVOL0C.
