# Supplementary material for: A methodological framework for developing bioinformatics databases: a case study on the Boesenbergia rotunda genome database
Source: Front Genet. 2026 Sep 9;17:1901079. doi: 10.3389/fgene.2026.1901079 (PMC13597081; doi:10.3389/fgene.2026.1901079)
Supplement: Supplementary file 1 [file DataSheet1.pdf]

Appendix

Table S1: The affected entries in swissprot_geneName.csv and trembl_geneName.csv

SP_geneName_ID	Subject_id_swissprot	Gene_name
1. SP001923	Q9SV13	1
2. SP003307	Q0D9S3	1
3. SP003309	A2YGP9	1
4. SP003888	Q5VND6	1
5. SP005101	Q9SDI4	1
6. SP008688	Q9SAY1	1
7. SP020636	O04722	1
8. SP021012	Q7XPF8	1
9. SP025659	Q9FY46	1
10. SP026981	Q7XU31	1
11. SP001925	O04289	2
12. SP003305	Q93XI5	2
13. SP003880	B8AW64	2
14. SP003882	Q53WK4	2
15. SP003884	Q9ZUP3	2
16. SP003886	Q70Z18	2
17. SP008690	Q9MAX3	2
18. SP009465	P10349	2
19. SP017132	Q53WP9	2
20. SP020639	P92946	2
21. SP025657	Q8GYH8	2
22. SP001927	Q9SXS2	3
23. SP003313	Q8L481	3
24. SP005104	Q8W0H5	3
25. SP008686	Q9FEP7	3
26. SP021010	Q6H501	3
27. SP025881	Q70Z17	3
28. SP001929	Q9LW86	4
29. SP002393	Q8GX78	4
30. SP002395	Q652N5	4
31. SP003311	Q8L4K5	4
32. SP003199	Q94LW6	5
33. SP024864	Q0IZQ3	5
34. SP002390	Q53P54	6
35. SP002388	Q2QWW7	7

Table S1 continued

SP_geneName_ID	Subject_id_trembl	Gene_name
1. TR02259	K0FGD5	1
2. TR02489	A0A077KP88	1
3. TR02891	G0YWA2	1
4. TR04291	A0A0B5WYA8	1
5. TR22755	G4XN34	1
6. TR23899	D2KZ47	1
7. TR24764	A7YGI3	1
8. TR24801	G8DRF9	1
9. TR26929	Q8GVD8	1
10. TR31943	Q4LDW9	1
11. TR40403	D2KZ44	1
12. TR49878	A8Y915	1
13. TR49880	G9BZV2	1
14. TR56242	A0A0A1G6J1	2
15. TR26343	Q0E3H2	3
16. TR42654	A0A0B5WYW1	3
17. TR02261	A0A0A1G395	4
18. TR02263	A0A0A1G6I7	5
19. TR27160	A0A0A1E5Q3	5
20. TR35709	Q1XGF1	5
